# Sleep disturbances and psychological well-being among military medical doctors of the Swiss Armed Forces: study protocol, rationale and development of a cross-sectional and longitudinal interventional study

**DOI:** 10.3389/fpubh.2024.1390636

**Published:** 2024-08-06

**Authors:** Dena Sadeghi-Bahmani, Viola Rigotti, Zeno Stanga, Undine E. Lang, Rebecca K. Blais, Michelle L. Kelley, Serge Brand

**Affiliations:** ^1^Department of Psychology, Stanford University, Stanford, CA, United States; ^2^Department of Epidemiology and Population Health, Stanford University, Stanford, CA, United States; ^3^University Hospital of Basel, Outpatient Medical Clinic, Basel, Switzerland; ^4^Centre of Competence for Military and Disaster Medicine, Swiss Armed Forces, Bern, Switzerland; ^5^Division of Diabetes, Endocrinology, Nutritional Medicine and Metabolism, University Hospital and University of Bern, Bern, Switzerland; ^6^Psychiatric Hospital of the University of Basel, Basel, Switzerland; ^7^Department of Psychology, Arizona State University, Tempe, AZ, United States; ^8^Department of Psychology, Old Dominion University, Norfolk, VA, United States; ^9^Psychiatric Hospital of the University of Basel, Center for Affective, Stress and Sleep Disorders, Basel, Switzerland; ^10^Division of Sport Science and Psychosocial Health, Department of Sport, Exercise and Health, University of Basel, Basel, Switzerland; ^11^Center of Military Disaster Psychiatry and Disaster Psychology, Psychiatric Hospital of the University of Basel, Basel, Switzerland

**Keywords:** sleep disturbances, Swiss Armed Forces, psychological well-being, military medical doctors, eCBTI, active control condition

## Abstract

**Background:**

Compared to civilians and non-medical personnel, military medical doctors are at increased risk for sleep disturbances and impaired psychological well-being. Despite their responsibility and workload, no research has examined sleep disturbances and psychological well-being among the medical doctors (MDs) of the Swiss Armed Forces (SAF). Thus, the aims of the proposed study are (1) to conduct a cross-sectional study (labeled ‘Survey-Study 1’) of sleep disturbances and psychological well-being among MDs of the SAF; (2) to identify MDs who report sleep disturbances (insomnia severity index >8), along with low psychological well-being such as symptoms of depression, anxiety and stress, but also emotion regulation, concentration, social life, strengths and difficulties, and mental toughness both in the private/professional and military context and (3) to offer those MDs with sleep disturbances an evidence-based and standardized online interventional group program of cognitive behavioral therapy for insomnia (eCBTi) over a time lapse of 6 weeks (labeled ‘Intervention-Study 2’).

**Method:**

All MDs serving in the SAF (*N* = 480) will be contacted via the SAF-secured communication system to participate in a cross-sectional survey of sleep disturbances and psychological well-being (‘Survey-Study 1’). Those who consent will be provided a link to a secure online survey that assesses sleep disturbances and psychological well-being (depression, anxiety, stress, coping), including current working conditions, job-related quality of life, mental toughness, social context, family/couple functioning, substance use, and physical activity patterns. Baseline data will be screened to identify those MDs who report sleep disturbances (insomnia severity index >8); they will be re-contacted, consented, and randomly assigned either to the eCBTi or the active control condition (ACC) (‘Intervention-Study 2’). Individuals in the intervention condition will participate in an online standardized and evidence-based group intervention program of cognitive behavioral therapy for insomnia (eCBTi; once the week for six consecutive weeks; 60–70 min duration/session). Participants in the ACC will participate in an online group counseling (once the week for six consecutive weeks; 60–70 min duration/session), though, the ACC is not intended as a *bona fide* psychotherapeutic intervention. At the beginning of the intervention (baseline), at week 3, and at week 6 (post-intervention) participants complete a series of self-rating questionnaires as for the Survey-Study 1, though with additional questionnaires covering sleep-related cognitions, experiential avoidance, and dimensions of self-awareness.

**Expected outcomes:**

Survey-Study 1: We expect to describe the prevalence rates of, and the associations between sleep disturbances (insomnia (sleep quality); sleep onset latency (SOL); awakenings after sleep onset (WASO)) and psychological well-being among MDs of the SAF; we further expect to identify specific dimensions of psychological well-being, which might be rather associated or non-associated with sleep disturbances.

Intervention-Study 2: We expect several significant condition-by-time-interactions. Such that participants in the eCBTi will report significantly greater improvement in sleep disturbances, symptoms of depression, anxiety, stress reduction both at work and at home (family related stress), and an improvement in the overall quality of life as compared to the ACC over the period of the study.

**Conclusion:**

The study offers the opportunity to understand the prevalence of sleep disturbances, including factors of psychological well-being among MDs of the SAF. Further, based on the results of the Intervention-Study 2, and if supported, eCBTi may be a promising method to address sleep disturbances and psychological well-being among the specific context of MDs in the SAF.

## 1 Introduction

Findings from cross-sectional and longitudinal studies demonstrated that restorative sleep is associated with a broad variety of health benefits, including emotional and cognitive benefits and specifically, less impulsivity, enhanced driving behavior in traffic, and better social interactions. By contrast, poor sleep is associated with symptoms of depression and anxiety (1–12), low impulse control (13, 14), non-suicidal self-injury and suicidal behavior (10, 12, 15–20), impaired cognitive and emotional processing (21–36), reckless or aggressive driving behavior (37–44), and impaired social behavior (45, 46).

For short, findings from cross-sectional and longitudinal studies among children, adolescents, and adults and from non-clinical and clinical samples consistently demonstrated that impaired sleep is associated with a broad range of psychological issues on well-being, including cognitive, emotional, and social behavior problems.

### 1.1 Sleep disturbances and psychological well-being among medical doctors

Medical doctors (MDs) are at increased risk of mental health issues due to low sleep quality, irregular sleeping schedules, increased latency in falling asleep and increased frequency of sleep interruptions (47–51). These issues may lead to symptoms of depression, anxiety (47–54) and burnout (54–59). To explain such impairments among MDs, research has identified long and irregular working hours, irregular sleep schedules (52, 60, 61) and stress (48, 54, 58, 59, 62–64) as main drivers associated with sleep disturbances. Given these associations, in the present study, our focus is on sleep disturbances and psychological well-being among MDs, though, among MDs in the context of the Swiss military (Swiss Armed Forces).

### 1.2 Sleep disturbances among military personnel

There is sufficient evidence that compared to non-military personnel, military personnel report more sleep disturbances, in that military personnel have specific and unique work-related demands and work and life style patterns (65). Bai et al. (66) reported a number of factors that may increase sleep disturbances, especially during military operations. These include frequent involvement in high-risk activities (67), context-specific standards (65), misalignments of stable sleep–wake-rhythms, such as a disruptive sleep environment, concerns about family issues back home (68), sustained operations, excessive caffeine intake, along with the exposure to physical and psychological injuries (69). Mantua et al. (70) investigated the associations between sleep patterns and high-risk behavior among 2,296 deployed military personnel (mean age 24.7 years) and concluded that sufficient and restorative sleep may decrease poor or risky decision making in the context of military duty.

Bai et al. (66) conducted a systematic review and meta-analysis of 59 studies with a total of 28,100 participants (Ns = 14 to 8,481) involving military and veteran participants. Bai et al. (66) calculated that the overall pooled prevalence of sleep disturbances in military personnel and veterans was 69.00% (95% CI: 62.33–75.30%); pooled rates were 57.79% (95% CI: 49.88–65.50%) for active-duty personnel, and 82.88% (95% CI: 74.08–90.21%) for veterans. Further, a higher mean age, and the occurrence of symptoms of depression and posttraumatic stress disorder (PTSD) were associated with higher prevalence rates of impaired sleep. Crane et al. (71) investigated the professional and private re-integration of military personnel after their military deployment and identified adequate and restoring sleep as a key variable causally related to a favorable post-deployment process. Further factors conferring to a more successful post-deployment process were: Lower scores for shame, guilt, and negative cognitive appraisal, along with a higher motivation to deploy successfully predicted a favorable outcome in the post-deployment phase.

### 1.3 Sleep disturbances among medical doctors in the military context

Regarding sleep disturbances among military MDs, research is scarce, and consistent data are lacking. Hsu et al. (72) observed that among 1,003 US Army physicians just 25% of respondents were adherent to the recommended 8 h sleep per day. When following the recommended 8 h sleep per day, about 28.4% reported a positive response to this regimen. Better sleep was associated with being a staff physician (compared to being a physician in training), and working fewer hours/week.

In a systematic review (73) summarizing 14 publications on military physicians’ sleep and professional performance, it turned out that sleep deprivation in any ‘out-of-hours’ surgery had a significant impact on overall morbidity and mortality. Sleep deprivation in surgeons and surgical trainees did negatively impact on their cognitive performance, putting their own and patients’ health at risk. More specifically, forward surgical teams will become combat-ineffective after 48 h of continuous operations (73). In a simulation study with 77 medical personnel of the Air National Guard (a federal military reserve force of the United States Air Force) it turned out that participants’ sleep quantity significantly decreased from civilian to disaster-training periods, while their cognitive performance decreased in parallel (74). In a sample of 344 medical staff members deployed to Afghanistan, they reported significantly higher levels of burnout and stress, compared to other professions (75). Adler et al. (75) did not specifically assess sleep quality or quantity; however, based on previous studies on the relation between poor sleep and increased stress (48, 62, 71, 76), we may assume that these 344 medical staff members would have reported higher scores for sleep disturbances.

Last, the National Guard (NG) served as critical component of the US COVID-19 response. A total of 3,221 NG service members were surveyed after their service in response of the COVID-19 (mean duration: 18.6 weeks). Over one-third reported changes in usual sleep: 33% described poor sleep quality, and 21% had troubles falling or staying asleep (77). This study is important in that it described the sudden shift from civil life to military life, and such sudden shifts are often observed among personnel of the SAF, in general, and among MDs of the SAF, more specifically.

Given this background, one aim of the present study was to investigate the prevalence rates of sleep disturbances (insomnia; disrupted sleep continuity) among MDs of the Swiss Armed Forces (SAF; see details below).

### 1.4 Treatment of insomnia with cognitive behavioral therapy

To treat insomnia, specifically tailored CBT-interventions were established and standardized (CBTi). After a standardized and one-day long CBTi intervention, individuals with insomnia with no further mental health issues and randomly assigned to the intervention improved in their insomnia scores (78), compared to a wait-list condition. Results from meta-analyses (79–85) showed that compared to wait list or control conditions, CBTi delivered to clinical and non-clinical samples of children, adolescents and adults was always superior.

In the same vein, randomized clinical trials, and systematic review and meta-analyses evidenced that online-delivered CBTi programs (eCBTi) were as effective as ‘real’ and face-to-face CBTi interventions (86–92).

As for now, we are unaware of any eCBTi intervention to improve sleep disturbances among MDs in the military context. Given this, Intervention-Study 2 aims at investigating the feasibility and impact of a standardized eCBTi intervention on MDs of the SAF, compared to an active control condition (ACC).

### 1.5 The specific context of the Swiss Armed Forces

In Switzerland, military service is mandatory for men. After basic training the most skilled recruits continue on a voluntary basis their training as cadre. After training in the non-commissioned officers’ school, the 15 weeks long officers’ school (OS) begins. This is considered to be stressful and demanding, both physically and mentally. The cadets are pushed to their limits every day during the extraordinarily intensive training. Importantly, male and female Swiss MDs in particular are encouraged to pursue the military career as military physicians.

Next, a unique aspect of the SAF is the high permeability between military and civil life. More specifically, throughout the basic military training, officers’ school, and during their annual courses, exercise trainings and refresher courses military personnel usually return home for weekends. Further, once an officer has achieved her/his rankings, annual courses and exercise trainings lasting for several days to up to 4 weeks per year are possible. At a behavioral and practical level, this implies that military MDs may switch between their private/professional and military deployments throughout their military service. Thus, the boundaries between private/civil/professional life and military life might get blurred, with quick and sometimes unpredictable transitions from civil/private/professional life to military deployments and vice versa. Such sudden changes may add to sleep disturbances. Until this date, we are unaware of studies on sleep disturbances, along with psychological well-being among MDs of the SAF. Given this, the present study aims at filling this gap of knowledge. Please consider that for the present study, exclusively medical doctors after their final exam (master’s degree) at a university were included, while officers, who did not yet complete the university degree as medical doctors were not considered.

### 1.6 The concepts of the transdiagnostic approach and of allostatic load

Within the last decade two psychiatric and cognitive-behavioral concepts have gained increased attention as explanations for why the effective treatment of a specific psychiatric disorder leads to improvements in other psychiatric conditions. To illustrate, treating symptoms of insomnia also improved symptoms of depression (93), anxiety, and stress (94–99). To explain this phenomenon, the concept of the *transdiagnostic approach* (97, 100–109) reflects the observation that improvements in one dimension of psychological experiences are associated with improvements in further dimensions of psychological experiences. Moreover, a meta-analysis on the treatment of anxiety disorders did not observe systematic differences between a disorders-specific cognitive-behavioral therapy (CBT) and a transdiagnostic CBT (tCBT) (108). Further, no associations between the comorbidity rate and tCBT outcome were observed. Likewise, Brand et al. (110) showed that acute bouts of physical activity impacted positively on mood, social interaction, and rumination among inpatients with psychiatric impairments, fully irrespective from patients’ psychiatric diagnosis.

Next, the concept of *allostatic load* (111–114) may help explain the improvements of non-specific benefits of CBT. Allostatic load is understood as the cumulative effects of stressful experiences in daily life, and such an alostatic load may lead to both a physiological and psychological strain over time. Thus, it is conceivable that improvements in sleep disturbances may improve symptoms of depression, anxiety and stress, in parallel.

### 1.7 The present study

Overall, there is sufficient evidence that compared to non-military personnel, military personnel report more impaired sleep, along with more issues of psychological well-being. This holds particularly true for MDs in the military context, though, research on this topic is scarce. More specifically, for MDs of the SAF, no research is available on the prevalence and association of sleep disturbances, along with concomitant issues of psychological wellbeing.

Given this background, the present study has the following aims:

Survey-Study 1: (1) To investigate the prevalence of sleep disturbances (insomnia; disrupted sleep continuity), and concomitant issues of psychological well-being among MDs of the SAF. (2) To investigate the patterns of associtions between sleep disturbances and issues of psychological well-being; (3) to identify MDs who report sleep disturbances (insomnia severity index >8), along with further dimensions of psychological ill-being and well-being such as symptoms of depression, anxiety, stress, emotion regulation, concentration, social activity, strengths and difficulties and mental toughness.

Intervention-Study 2: (3) To offer a stanrdardized online CBT program for insomnia (eCBTi). To this end, MDs reporting sleep disturbances will be randomly assigned either to the internvention condition (see details below) or to the active control condition (see details below).

For the Survey-Study 1, the hypothesis is: Higher scores for sleep disturbances are associated with more issues of psychological wellbeing such as depression, anxiety, and stress, including also social behavior.

For the Intervention-Study 2, the hypothesis is: Compared to the baseline and to an ACC, at the end of the intervention participants of the eCBTi report improved sleep disturbances, along with improved psychological well-being, as mentioned above.

## 2 Methods

### 2.1 Participants

We expect that all 480 MDs of the SAF complete the survey. The inclusion criteria are: 1. Aged 18 years and older; 2. Master degree or higher in human medicine; 3. MD of the SAF; 3. Willing and able to comply with the study conditions; 4. Signed written informed consent. Exclusion criteria are: 1. Completing the questionnaires in an unreliable fashion (“clickthroughts”; answers are given clicking systematically on the right or left side or in the middle; questionnaires are completed within a couple of minutes). 2. A participant withdraws from the study. 3. Pregnancy or breastfeeding, as this may alter sleep and psychological well-being. 4. A participant leaves the SAF.

### 2.2 Procedure

For this cross-sctional and interventional study all MDs of the SAF will be contacted via the secure e-mail system of the SAF electronic platform. The invitation e-mail will explain the study aims, the approval process, the voluntary nature of participation, and the coded data handling. Further, participants will be informed that participation or non-participation will have no advantages or disadvantages for their professional and military career. The study will be conducted according to the seventh and current version (115) of the Declaration of Helsinki, and the study has been approved on March 28, 2024 by the local ethics committee (EKNZ; Ethikkommission Nordwest- und Zentralschweiz; Basel, Switzerland: register code: 2024–00258).

### 2.3 Survey-study 1; study design

Participants who provide written informed consent will receive a survey link (see Table 1 for a description of the study measures). The baseline survey data will be analyzed to get a descriptive overview, including prevalence rates of participants’ sleep disturbances and psychological well-being. Next, those MDs with sleep disturbances along with issues of psychological well-being will be identified and are eligible for the Intervention-Study 2.

**Table 1:** Overview of the questionnaires.

Survey		
Dimension	Measure	Reference
Sociodemographic and military-related information	Self-administered questionnaire	
Sleep		
Insomnia	Insomnia Severity Index (ISI)	Gerber et al. (116) and Bastien et al. (117)
Sleep continuity	Pittsburgh Sleep Quality Index (PSQI; modified)	Buysse et al. (118)
		
Physical activity	International Physical Activity Questionnaire (IPAQ); short form	Craig et al. (119) and Lee et al. (120)
Psychological well-being		
Depression	Patient Health Questionnaire (PHQ-9)	Kroenke et al. (121)
Coping strategies	Stressverarbeitungsfragebogen [Coping with stress questionnaire]; short form	Erdmann and Janke (122)
Depression, anxiety, stress	Depression, anxiety, stress questionnaire (DASS-21)	Lovibond and Lovibond (123, 124)
Emotion regulation	Emotion Regulation Questionnaire	Gross and John (125)
Concentration	Adult Concentration Inventory	Fredrick et al. (126) and Sadeghi-Bahmani et al. (127)
Social activity	Social Adaptation Self-evaluation Scale (SASS)	Bech et al. (128)
Strengths and Difficulties	Strengths and Difficulties Questionnaire (SDQ); adult version	Goodman (129)
Mental toughness	Mental toughness Questionnaire; short-form, 18	Dagnall et al. (130)

Intervention					
Dimension	Measure	Reference		Time points	
			Baseline	Week 3	Week 6 (stud end)
Sociodemographic and military-related information	Self-administered questionnaire		X		
Sleep					
Insomnia	Insomnia Severity Index (ISI)	Bastien et al. (117) and Gerber et al. (116)	X	X	X
Sleep continuity	Pittsburgh Sleep Quality Index (PSQI; modified)	Buysse et al. (118)	X	X	X
Sleep-related cognitions and emotions					
**Dysfunctional beliefs and attitudes about sleep**	Dysfunctional beliefs and attitudes about sleep	Morin et al. (131)	X	X	X
**Pre-sleep cognitions and emotions, including bed time procrastination**	Bedtime Procrastination Scale (BPS)	Kroese et al. (132) and Kroese et al. (133)	X	X	X
					
Physical activity	International Physical Activity Questionnaire (IPAQ); short form	Craig et al. (119) and Lee et al. (120)	X		X
Psychological well-being					
Depression	Patient Health Questionnaire (PHQ-9)	Kroenke et al. (121)	X	X	X
Coping strategies	Stressverarbeitungsfragebogen [Coping with stress questionnaire]; short form	Erdmann and Janke (122)	X		X
Depression, anxiety, stress	Depression, anxiety, stress questionnaire (DASS-21)	Lovibond and Lovibond (123, 124)	X	X	X
Emotion regulation	Emotion Regulation Questionnaire	Gross and John (125)	X		X
Concentration	Adult Concentration Inventory	Fredrick et al. (126) and Sadeghi-Bahmani et al. (127)	X		X
Social activity	Social Adaptation Self-evaluation Scale (SASS)	Bech et al. (128)	X		X
Strengths and Difficulties	Strengths and Difficulties Questionnaire (SDQ); adult version	Goodman (129)	X		X
Mental toughness	Mental toughness Questionnaire; short-form, 18	Dagnall et al. (130)	X		X
**Experiential avoidance**	Experiential Avoidance Scale	Hayes et al. (134) and Zakiei et al. (99)	X	X	X
**Mindfulness self-awareness**	Freiburg Mindfulness Inventory (FMI)	Walach et al. (135)	X	X	X

Information in bold characters = additional questionnaires for the intervention study. X = completing the questionnaires.

### 2.4 Intervention-study 2; study design

Medical doctors reporting sleep disturbances (see above) are contacted and invited to participate at the intervention of eCBTi. They are informed about the study design, the aims of the intervention study, the coded data handling, along with the information that participation or non-participation will have no advantages or disadvantages for their military career. Next, participants are randomly assigned either to the eCBTi condition (see details below) or to the ACC (see details below). All participants complete a series of self-rating questionnaires (see details below) at baseline, at week 3 (half time) and at week 6 (study end). At the end of the intervention, participants in the ACC can switch to the eCBTi condition.

### 2.5 Measures

Survey-Study 1: Table 1 provides the overview of the study measures. The primary outcome measures are sleep disturbances (insomnia; sleep onset latency (SOL); awakenings after sleep onset (WASO) = disrupted sleep continuity). Secondary outcomes are psychological ill-being and well-being, including depression, anxiety, stress, coping strategies, emotion regulation, concentration, social activity, strengths and difficulties, and mental toughness.

Intervention-Study 2: Table 2 provides the overview of the study measures. Primary outcomes are change in sleep disturbances (insomnia; disrupted sleep continuity). Secondary outcomes are psychological ill-being and well-being, including the dimensions metioned above, and additionally: Sleep-related cognitions, experiential avoidance, and mindfulness self-awareness (see also Table 1).

**Table 2:** Elements of electronically delivered cognitive behavioral therapy for insomnia (eCBTi).

Session	Content
One	What is sleep? Provides an overview of the course and introduces the concepts of sleep, CBT, and insomnia.
Two	How various sleep habits impact on sleep. Provides stimulus control instructions, such as activities that are permitted or disallowed when in bed, daytime napping and what to do if sleep is not attained at night. Provides sleep restriction instructions, including setting specific bedtime, maintaining the same wake time, and restricting time spent in bed awake.
Three	Sleep hygiene education and discusses behavioral health practices, such as caffeine, alcohol, exercise, diet, smoking, and environmental factors’ impacts on sleep. Overview of relaxation techniques that support sleep.
Four	Role of automatic and negative thoughts and how they influence feelings. Identification of negative thoughts and alternative interpretations (cognitive restructuring)
Five	As session four; cognitive restructuring
Six	Review; summary of the main concepts of CBTi; what did I learn?

CBTi, cognitive behavioral therapy for insomnia.

#### 2.5.1 Online survey

Please consider that there will be a questionnaire for private/civil life and one for military deployment.

##### 2.5.1.1 Sociodemographic and military-related information

Participants report on their age, sex at birth, current job position (residency status; specialty; position within a hospital or extra-hospital activity); current military ranking and duration of military service (years).

##### 2.5.1.2 Sleep

To assess sleep dimensions, three questionnaires are used.

###### 2.5.1.2.1 Insomnia

Participants complete the German version (116) of the Insomnia Severity Index [ISI; (117)]. The questionnaire consists of seven items that assess difficulty falling asleep, sleeping without interruption, feeling refreshed in the morning, and daytime performance, in the last 2 weeks. Participants rate how well each item describes themselves from: 0 (= not at all) to 4 (= almost always), with higher sum scores reflecting a higher degree of insomnia.

###### 2.5.1.2.2 Sleep continuity

To assess sleep continuity of the last 2 weeks, participants will be administered the modified Pittsburgh Sleep Quality Inventory (PSQI (118):). The questionnaire assesses usual bedtime, sleep onset latency, the number of awakenings after sleep onset, and wake-up time.

###### 2.5.1.2.3 Sleep-influencing factors and rituals

The self-administered questionnaire evaluates working and sleep time, factors influencing sleep such as pets, children, on-call service, co-sleeping, noise and similar. Typical pre-sleep rituals are reading books, physical activity, texting/messaging, using tablets or similar electronic devices, sex, substances such as alcohol, cannabis, medications. Items are answered “yes” (scored as 1) or “no” (scored as 0.), with higher sum scores reflecting more unfavorable sleep-influencing factors.

##### 2.5.1.3 Physical activity

Participants complete the 7-item International Physical Activity Questionnaire-Short Form (119) to assess weekly sedentary life style, and light to moderate and vigorous physical activity patterns. An overall total physical activity MET-minutes/week score will be calculated, and according to the amount of physical activity the subjects will enter the category of low, moderate or high physical activity.

##### 2.5.1.4 Depression

To assess symptoms of depression, participants will be asked to complete the PHQ-9 (122), a brief depression severity measure; higher overall summed scores indicate higher symptoms of depression (0–4 points indicate no depression, 5–9 mild depression, 10–14 moderate depression, 15–19 moderatly severe and 20–27 severe depression).

##### 2.5.1.5 Coping strategies

To assess coping strategies, participants will be administered the short-form of the Stressverarbeitungsfragebogen 120 [Coping with stress questionnaire] (121). Favorable coping strategies are: Minimizing and trivializing, self-instructions, active problem solving. Unfavorable coping strategies are: Social withdrawal, catastrophizing, substance use. Higher summed scores reflect more pronounced favorable and unfavorable coping strategies.

##### 2.5.1.6 Depression, anxiety, stress

Participants will complete the Depression, Anxiety, Stress-questionnaire [DASS-21; (123, 124)]. Higher summed scores indicate a higher degree of depression, anxiety and stress.

##### 2.5.1.7 Emotion regulation

Participants will complete the Emotion Regulation Questionnaire (125), which focuses on cognitive reappraisal and emotion suppression. Item scores will be summed to create a total score; higher overall scores indicate more cognitive reappraisal and more emotion suppression.

##### 2.5.1.8 Concentration

Participants complete the German version (Sadeghi-Bahmani and Brand, in preparation) of the Adult Concentration Inventory (126, 127); higher sum scores reflect more difficulties with concentration.

##### 2.5.1.9 Social activity

Participants will complete the Social Adaptation Self-evaluation Scale [SASS; (128)]. Higher scores indicate higher social activity.

##### 2.5.1.10 Strengths and difficulties

Participants will complete the Strength and Difficulties Questionnaire [SDQ; (129)] for adults. Dimensions are: Emotional problems, behavioral problems, hyperactivity, problems with peers and family members, and prosocial behavior.

##### 2.5.1.11 Mental toughness

Participants complete the mental toughness short form [MTQ-18; (130, 136)], with higher scores reflecting a higher degree of mental toughness.

#### 2.5.2 Measures for the eCBTi intervention and active control condition

The following questionnaires will be identical in both the initial survey and the intervention study: Sociodemographic and military-related information, sleep, physical activity, depression, coping strategies, depression, anxiety and stress, emotion regulation, social activity, strengths and difficulties, mental toughness (see Table 1).

In addition to the above mentioned questionnaires, the intervention study will also assess several additional psychological constructs with the following surveys:

##### 2.5.2.1 Dysfunctional beliefs and attitudes about sleep

The Dysfunctional Beliefs and Attitudes about Sleep (DBAS; 16-items version) assesses cognitions and attitudes unfavorably impacting on sleep. Typical items are: “I need 8 h of sleep.,” or “It’s better taking sleeping pills.,” or “Sleep is unpredictable.” Answers are given on five-point Likert scales ranging from: 1 (= strongly disagree) to 5 (= strongly agree), with higher sum scores reflecting a more pronounced tendency to display dysfunctional beliefs and attitudes about sleep.

##### 2.5.2.2 Pre-sleep cognitions and emotions, including bed time procrastination

The Bedtime Procrastination Scale [BPS (132, 133)] consists of nine items measuring subjective bedtime procrastination. Typical items are: “I easily get distracted by things when I actually would like to go to bed,” “I go to bed later than I had intended,” and “I want to go to bed on time but I just do not.” The response scale ranges from 1 (= almost never) to 5 (= almost always), with higher sum scores reflecting a higher tendency to delay to going to bed and to sleep.

##### 2.5.2.3 Experiential avoidance

Participants will be administered the Experiential Avoidance Questionnaire [Hayes et al. (99, 137)]. It consists of 10 items, and typical items include: “I am afraid of my feelings.” or “My thoughts and feelings mess up my life.” Answers are given on 7-point Likert scales ranging from 1 (= never) to 7 (= always). Higher sum scores reflect a higher degree to accept also unpleasant experiences (emotions, cognitions), and accordingly, a lower degree of avoidance.

##### 2.5.2.4 Mindfulness – self-awareness

Participants complete the Freiburg Mindfulness Inventory (FMI) (135). The short form consists of six items. Sample items are: “I perceive my feelings and emotions without having to react to them,” “I observe how my thoughts come and go,” “I accept unpleasant feelings,” and “I’m able to smile when I notice how I sometimes make life difficult.” Items are rated on a 4-point scale from 1 (= rarely) to 4 (= almost always), with higher sum scores reflecting a higher tendency of mindfulness and self-awareness.

### 2.6 Interventions

#### 2.6.1 eCBTi

We follow the standardized and validated intervention protocol for eCBTi (87, 91, 92; see Table 2). The intervention lasts for six consecutive weeks, with one session per week; a session lasts between 60 min to 70 min. Weekly homeworks need about 60–90′ per week.

#### 2.6.2 Active control condition

As described extensively elsewhere (99, 138–141) participants in the control condition will meet once a week for six consecutive weeks for about 70 min. Thus, frequency, duration, and intensity are identical to the intervention condition. For homework, participants are asked to journaling. The control condition could not be considered as a *bona fide* intervention, given that the sessions exclude treatment elements that are truly intended to be therapeutic (142). Rather, participants will have group discussions on daily activities and daily problems. Participants are encouraged to exchange daily life experiences. The active control condition is not intended to be an active therapy, but to control for possible placebo effects in the intervention condition.

Licensed and trained clinical psychologists are responsible for both the eCBTi and the ACC.

### 2.7 Sample size calculations

To calculate the sample size for the intervention study, we used the G*Power software (143). Based on previous studies on the efficacy of eCBTi (86–92), we expect a partial eta-squared of 0.08 (medium effect); alpha: 0.05; Power: 0.95; number of groups: 2; number of measurements: 3; total sample size: 32, that is, 16 per condition. To counterbalance possible drop-out, we assess 25 participants per condition.

### 2.8 Analytical plan

Survey-Study 1: We will examine the prevalence rates of insomnia (continuous and categorical dimensions), disrupted sleep continuity, physical activity, psychological well-being, including depression, anxiety, stress, coping with stress, emotion regulation, concentration, social activity, and mental toughness. A series of Pearson’s correlations are run to calculate the associations between the variables mentioned above. Chi-square-tests will be used to calculate the associations between categorical dimensions. To explore which psychological dimensions are statistically more strongly associated with insomnia, we run regression analyses; the minimum requirements are as follows (137, 144, 145): *N* = x > 100; predictors explain the dependent variables (R > 0.40, R^2^ > 30); the number of predictors x 10 should be < N; the Durbin–Watson coefficients should be between 1.5 and 2.5, indicating that the residuals of the predictors are independent. For the variances inflation factors (VIF): While there are no strict cut-off points to report the risk of multicollinearity, VIF < 1 and VIF > 10 indicate multicollinearity.

For t-tests, Cohen’s d effect sizes are reported with the following cut-off values: Trivial (ds: 0–0.19), small (ds: 0.20–0.49), medium (ds: 0.50–0.79) or large (ds: 0.80 and greater) (146–148).

#### 2.8.1 Intervention

ANOVAs for repeated measures are run with the factors Group (eCBTi vs. ACC), Time (baseline, week 3, week 6), and the Group x Time-interaction; dependent variables as reported in Table 2. Post-hoc analyses after Bonferroni-Holm are used for p-corrections for multiple testing. Single mean comparisons are performed with Cohen’s d effect sizes, as proposed by Becker (149). In case of missing values, such missing data will be replaced with the means or medians of the sample (144, 145, 150, 151). For the intervention study: Statistical procedures are run both by protocol (that is: only available data observed at any time point from every participant are considered) and by intent-to-treat (ITT) with the last intervention carried forward (LOCF) (152). Further, we consider the following confounders, that is to say, we statistically calculate, whether the pattern of results might get biased in the same or opposite directions for the following confounders: Age, gender, working condition, including current full time or part time positions as medical doctors, including their subspeciality such as neurosurgeon, anesthesiologist, or emergency MD, to name but a few, and military rank, or time lapse between the assessment and the last deployment.

### 2.9 Assessment of safety

Survey: Completing the online survey has no risks of adverse effect. However, at the beginning, in the middle, and at the end of the booklet we provide websites, email-addresses and phone numbers of local emergency units for psychiatric issues.

Intervention: To our understanding, no adverse effects or risks are reported for eCBTi interventions. As such, also in previous studies CBTi interventions are considered as low-risk psychotherapeutic interventions (87, 92, 153).

### 2.10 Data protection

First, for both the Survey and the Internvention study, all participants sign the written informed consent, which clearly warrants full privacy and fully data protection. More specifically, neither the ranking officers of the SAF nor anybody else has access to the data. In line with this, the data are securely stored on a cluster of the server of the Psychiatric Hospital of the University of Basel (UPK, Basel, Switzerland). As such, no one except the study coordinator and some very specifically trained psychologists of the UPK who are by no means associated with the SAF, have access to the data set. Further, all data are coded, and the code-key is securely and separately stored from the data sheet. These circumstances warrant full anonymity and data protection, which are particularly important and delicate in organizations and systems such as the military.

### 2.11 Status and time line

The present study protocol has been approved on March 28, 2024 by the local ethics committee (EKNZ; Ethikkommission Nordwest- und Zentralschweiz; Basel, Switzerland: register code: 2024–00258). The timeline is reported in Table 3.

**Table 3:** Time table of both studies.

Time (months)	July	July/August	September	September 2024 to February 2025	February to May 2025
All medical doctors of the SAF are contacted	Information	Screening	1st visit	2nd visit	
Written Information	+				
Written consent	+				
Link to the online survey		+			
Reminder to participate		+			
Survey closed			+		
Contacting people interested in the intervention			+	+	
Written Information			+	+	
Written consent			+	+	
Group intervention of eCBTi (small groups; 6 weeks); active control condition in parallel (ACC)			+	+	
Former participants of the ACC switch to the eCBTi			+	+	
Data analysis and first publications					+

## 3 Discussion

Sleep issues have become a major health concern in the general population. Further, compared to the general population, two subgroups are at increased risk to report poor sleep: Medical doctors (47–51), and military personnel (65, 66, 154). The logic is that military MDs should be at additional high risk to suffer from sleep issues. To our understanding, such data are missing in general, and for the Swiss Armed Forces (SAF), in specific. To counter this, the present study consists of two parts:

Survey-Study 1: The first part will be the survey with the aim to assess sleep disturbances (insomnia; disrupted sleep continuity), including dimensions of psychological well-being among military MDs of the SAF. Based on the results gathered during this survey, the second part starts, that is:

Intervention-Study 2: Participants reporting poor sleep (Insomnia Severity Index >8), including poor psychological well-being, and interested in undergoing a standardized and validated treatment for insomnia (eCBTi) (87, 91, 92) are randomly assigned either to the intervention condition (eCBTi) or to the active control condition (ACC). The interventions last six consecutive weeks, with weekly group sessions lasting for 60–70 min, plus weekly homework. At the end of the intervention, participants in the ACC have the possibility to switch to the eCBTi intervention.

### 3.1 Expected outcomes: survey-study 1

We expect to gather data on the prevalence of sleep disturbances among MDs of the SAF, both in their private/professional and military context. We further should understand, if and to what extent sleep disturbances are further associated with psychological dimenions of psychological well-being and ill-being, including depression, anxiety, stress, coping with stress, emotion regulation, concentration, social activity, strength and difficulties, and mental toughness. Last, we expect to indentify those MDs reporting sleep disturbances and being willing and able to undergo a standardized psychotherapeutic intervention of eCBTi.

#### 3.1.1 Expected outcomes: intervention-study 2

We expect that compared to individuals in the ACC, and compared to the baseline, at the end of the intervention, individuals in the eCBTi condition report improved sleep disturbances, including dysfunctional cognitions impairing sleep, along with improvements for symptoms of depression, anxiety, coping with stress, emotion regulation, concentration, social activity, strength and difficulties, and mental toughness, and with improvements for experiential avoidance and mindfulness self-awareness. The intervention study should also help to understand possible improvements in sleep disturbances and psychological well-being both in the professional/private and in the military context.

### 3.2 Limitations

We are aware of the following study limitations. First, all data are self-reported, and as such, biases in the self-perception cannot be ruled-out. Ideally, psychiatrists and clinical psychologists would run clinical interviews to assess possible psychiatric disorders (155, 156). Second, we assess sleep disturbances, including psychological well-being both in a participants’ military and family/private/professional context: While the unique structure of SAF military service, including repeated regular training courses over several years, helps to keep an expert’s specific military skills high, the boundaries between civil/professional and military life might get blurred, with quick and sometimes unpredictable transitions from civil life to military deployments and vice versa. As such, it might be challenging to complete the questionnaires, while cognitively discerning between civil/professional and military life. Third, given the strictly voluntary character of both the survey and the intervention and considering a MD’s workload, it is not clear, how many military medical doctors will participate. Fourth, very similar to the literature on sleep disturbances among military personnel and military MDs (see Introduction section), we mainly assess insomnia and disrupted sleep continuity, while further sleep disturbances such as Restless Legs Syndrome (RLS), Obstructive Sleep Apnea (OSA), parasomnias, including night terrors and night mares are not considered. However, one paragraph in the survey asks more specifically about sleep-impairing events; as such, participants could report sleep-issues such as RLS, OSAs, and parasomnias. Fifth, we focused prevalently on sleep quality (insomnia), while sleep quantity (disrupted sleep continuity such as delayed sleep onset latency and awakenings after sleep onset) was regarded as less important (157).

## Author contributions

DS-B: Conceptualization, Methodology, Project administration, Supervision, Validation, Visualization, Writing – original draft, Writing – review & editing, Data curation. VR: Conceptualization, Project administration, Writing – original draft, Writing – review & editing. ZS: Conceptualization, Writing – original draft, Writing – review & editing, Funding acquisition, Investigation, Methodology, Resources, Supervision. UL: Conceptualization, Methodology, Resources, Writing – original draft, Writing – review & editing. RB: Conceptualization, Methodology, Resources, Writing – original draft, Writing – review & editing, Supervision. MK: Conceptualization, Methodology, Resources, Supervision, Writing – original draft, Writing – review & editing. SB: Conceptualization, Methodology, Resources, Supervision, Writing – original draft, Writing – review & editing, Formal analysis, Funding acquisition, Investigation, Project administration, Software, Validation, Visualization.
